# Laryngeal mask placement in a teaching institution: analysis of difficult placements

**DOI:** 10.12688/f1000research.6415.1

**Published:** 2015-04-29

**Authors:** Anastasia D Katsiampoura, Peter V Killoran, Ruggero M Corso, Chunyan Cai, Carin A Hagberg, Davide Cattano

**Affiliations:** 1 Department of Anesthesiology, University of Texas Medical Science Center, Houston, TX, USA; 2 Emergency Department, Anesthesia and Intensive Care Section, “GB Morgagni-L.Pierantoni” Hospital, Forli, 47121, Italy; 3 Division of Clinical and Translational Sciences, Department of Internal Medicine, University of Texas Medical Science Center, Houston, TX, USA

**Keywords:** airway management, LMA, anesthesia, neck circumfrence

## Abstract

**Background**: Laryngeal mask airway (LMA) placement is now considered a common airway management practice. Although there are many studies which focus on various airway techniques, research regarding difficult LMA placement is limited, particularly for anesthesiologist trainees. In our retrospective analysis we tried to identify predictive factors of difficult LMA placement in an academic training program.

**Methods**: This retrospective analysis was derived from a research airway database, where data were collected prospectively at the Memorial Hermann Hospital, Texas Medical Center, Houston, TX, USA, from 2008 to 2010. All non-obstetric adult patients presenting for elective surgery requiring general anesthesia, were enrolled in this study: anesthesiology residents primarily managed the airways. The level of difficulty, number of attempts, and type of the extraglottic device placement were retrieved.

**Results**: Sixty-nine unique Laryngeal Mask Airways (uLMAs) were utilized as a primary airway device. Two independent predictors for difficult LMA placement were identified: gender and neck circumference. The sensitivity for one factor is 87.5% with a specificity of 50%. However with two risk factors, the specificity increases to the level of 93% and the sensitivity is 63%.

**Conclusion**: In a large academic training program, besides uLMA not been used routinely, two risk factors for LMA difficulty were identified, female gender and large neck circumference. Neck circumference is increasingly being recognized as a significant predictor across the spectrum of airway management difficulties while female gender has not been previously reported as a risk factor for difficult LMA placement.

## Introduction

Since its introduction into clinical practice in 1983 ^1^, the laryngeal mask airway (LMA) has found a place in everyday anesthesia practice ^2– 4^, including its use as a primary airway device in the elective or pre-hospital emergency settings, as well as a rescue airway device in either settings ^5, 6^. Additionally, the LMA placement has become a common airway management technique, particularly in ambulatory surgery ^2, 3^, and is associated with shorter recovery time, earlier patient discharge and lower associated costs ^7, 8^. Even if the LMA is considered a very safe airway device ^9^ with a low incidence of complications, there may be situations where it either does not function properly or is difficult to place ^10^. Importantly, the association between difficult LMA placement and increased incidence of Difficult Mask Ventilation (DMV) has been recognized ^11^.

Appropriate sizing is critical for correct LMA application ^12^, while the selection of the device type seems to play a less significant role, yet the prediction of the correct size is not easy. This can be attributed to the absence of a coherent and universal standard sizing system ^13^. Most of the manufacturers suggest a weight-based size selection, however there is no consistency between weight and oropharyngeal anatomy ^14^.

Alternative recommendations for the selection of the appropriate size of a LMA, regarding age, height and gender, as well as anatomical landmarks, are still under investigation ^15– 17^.

As a result, the concepts of difficult LMA placement and effective usage have prompted new research, focusing on the prediction of difficult LMA placement ^18^.

A simple, objective, predictive score to identify patients at risk of difficult LMA placement at the bedside does not currently exist, however to achieve such score a comprehensive airway assessment based analysis of risk identification needs to be accomplished first. Based on recorded outcomes at a major teaching hospital that utilized a comprehensive airway assessment ^19^ we aimed to identify predictive factors for difficult LMA placement.

## Methods

Data for this retrospective analysis were derived from a database of airway assessments, management plans, and outcomes collected prospectively from August, 2008 to May, 2010 at a Level 1 academic trauma center (Memorial Hermann Hospital, Texas Medical Center, Houston, TX, USA) ^11^. The study was sponsored by an educational grant from the Foundation for Anesthesia, Education and Research (FAER), and other educational funds from the Department of Anesthesiology at University of Texas Medical School at Houston. After obtaining IRB approval, (HSC-MS-07-0144) all non-obstetric adult patients presenting for elective surgery requiring general anesthesia were enrolled in this study (n=8364). All uLMA placements were carried out by anesthesiology residents. In the ‘mother study’, residents were randomized into two groups—an experimental group, which used a comprehensive airway assessment form ^11, 20^ in addition to the existing anesthesia record, and a control group, which used only the existing anesthesia record. For the purpose of the present analysis, only the experiment (n=2348) group data was utilized, since the comprehensive airway assessment needed to be linked to the airway device that was utilized. We identified 110 cases-used of LMA, disposable laryngeal mask (uLMA, North America, San Diego, CA), and 69 of those as primary airway device, which we utilized for our analysis. Difficult LMA placement was defined as either inability to physically place a LMA device or inadequacy of ventilation, oxygenation, or airway protection after placement that required conversion to an alternative technique. The level of difficulty and the number of attempts of the uLMA placement were documented by the anesthesiology residents.

## Statistical analysis

**Figure 1.**
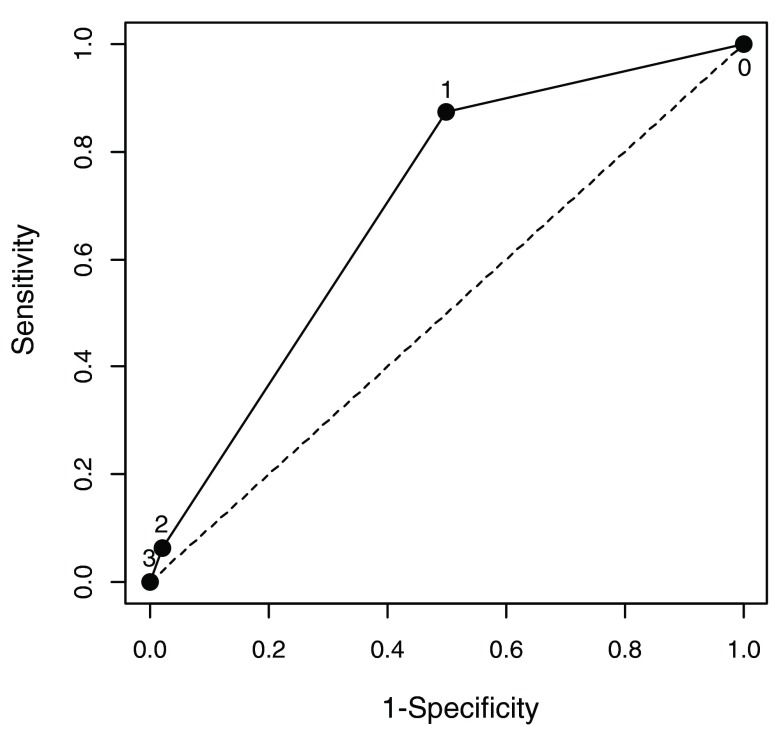
A receiver-operating-characteristic (ROC) curve evaluating the sensitivity and specificity of preoperative independent risk factors for LMA difficulty. Two independent predictors for LMA difficulty were identified using logistic regression: Female and NeckCirc of 44 or greater. The area under the curve was 0.69. The area under the curve was calculated to evaluate the resulting model’s predictive value. The adjusted odds ratios and their 95% confidence interval were calculated. Continuous variables were included after the dichotomization and the best cut-off was determined by maximizing the sum of sensitivity and specificity using the ROC curve. Analyses were conducted using SAS 9.3 (SAS Institute, Cary, NC, USA).

Sixty nine uLMA placements were completed and an analysis was performed (based on “per protocol” and not intention to treat). The mean and standard deviation were used to summarize continuous variables, and frequency (percentage) was summarized for categorical variables. A two-tailed sample t-test was applied to compare continuous variables and Chi-square or Fisher exact tests as appropriate were performed for categorical variables between patients with or without uLMA placement difficulty. Using multivariate logistic regression models, the variables associated with uLMA placement difficulty were identified. All variables with a p-value ≤0.25 in univariate analysis and variables of known biological importance (e.g., age and BMI) were entered into a full model. A backward selection method was used to identify significant independent predictors. A receiver-operating-characteristic (ROC) area under the curve was also calculated to evaluate the resulting model’s predictive value, ( Figure 1) as well as adjusted odds ratios and their 95% confidence intervals. Continuous variables were included after the dichotomization and the best cut-off was determined by maximizing the sum of sensitivity and specificity using the ROC curve. Age distribution for our population was assessed by using descriptive statistics including mean, standard deviation, and median values. All statistical analyses were conducted using SAS 9.3 (SAS Institute, Cary, NC, USA). A p-value <0.05 was considered significant.

## Results

Patient demographics are presented in Table 1 and Table 2. Of the airway evaluations performed using a comprehensive airway assessment tool 69 LMAs were utilized as a primary airway device ( Table 3). Of these, 67 were successful (97.1%) and 2 were unsuccessful (2.9%), with 17 (24.6%) uLMA placements considered as difficult ( Table 4). Multivariate logistic regression models identified two independent predictors of difficult airway: gender and neck circumference ( Table 5). The risk of difficult LMA placement was significantly higher for female patients and patients with a neck circumference (≥44 cm). The model’s c-statistic score is 0.69 ( Table 6). When at least one of two identified risk factors as a cut-off for predicting difficult LMA placement is present, the sensitivity is 87.5% and the specificity is 50%. If we use two risk factors as a cut-off, the specificity increases to the level of 98% and sensitivity is 63% ( Table 5).

**Table 1.** Preoperative patient characteristics by LMADiff status.

Variables	LMADiff		p-value
	False (LMADiff=0) N=52	True (LMADiff=1) N=17	
**Age (year)**, mean±SD <35, n (%)	48±19 ^1^ 17 (33.3)	51±16 2 (11.8)	0.608 0.121
**Male**, n (%)	33 (64.7) ^1^	8 (47.1)	0.198
**Height (cm)**, mean±SD <175, n (%)	172.8±10.9 ^3^ 23 (46.9)	169.1±8.5 12 (70.6)	0.206 0.092
**Weight (kg)**, mean±SD	79.9±16.0	78.9±23.9	0.870
**BMI (kg/m ^2^)**, mean±SD <30, n (%)	26.9±5.8 ^1^ 39 (81.3)	27.5±8.2 ^2^ 12 (70.6)	0.744 0.493
**Neck Circumference**, mean±SD <44, n (%)	39.3±4.3 ^1^ 43 (84.3)	40.0±6.6 ^4^ 10 (62.5)	0.686 0.082
**InterIncisors distance**, mean±SD	4.4±0.8	4.3±1.0	0.515
**Thyromental distance**, mean±SD	8.9±1.5 ^1^	9.1±0.9 ^4^	0.561
**Sternomental distance**, mean±SD	16.2±2.4 ^1^	16.1±2.1 ^4^	0.835
**Neck Mobility Grade**, n (%) 1 2,3	36 (69.2) 16 (30.8)	13 (76.5) 4 (23.5)	0.568
**Mallampati**, n (%) I, II III, IV	n=51 32 (62.8) 19 (37.3)	n=17 9 (52.9) 8 (47.1)	0.474
**U BiteTest** A B C	41 (78.9) 10 (19.2) 1 (1.9)	10 (58.8) 6 (35.3) 1 (5.9)	0.245
**Cervical Spine Abnormality**, n (%)	3 (5.8)	2 (11.8)	0.591
**NoTeeth**, n (%)	7 (13.5)	4 (23.5)	0.445
**Facial Hair**, n (%)	11 (21.2)	4 (23.5)	1.0
**Facial Trauma**, n (%)	1 (1.9)	2 (11.8)	0.148
**Nasal Defect**, n (%)	3 (5.8)	0 (0)	NR
**Neck Trauma**, n (%)	2 (3.9)	0 (0)	NR
**Short Neck**, n (%)	1 (1.9)	2 (11.8)	0.148
**Obstructive Sleep Apnea**, n (%)	25 (48.1)	8 (47.1)	1.0
**Thyroid**, n (%)	2 (3.9)	1 (5.9)	1.0

^1^N=51; ^2^N=14; ^3^N=49; ^4^N=16; NR: not reported due to zero cells; p-values are obtained by two sample t-test for continuous variables and Chi-square test or Fisher’s exact test as appropriate for categorical variables

**Table 2.** Age distribution of our population.

Gender	Age	
	mean±SD	Median (min, max)
Female (N=27)	51.1±17.4	53 (18, 79)
Male (N=41)	47.3±18.1	50 (20, 80)
All population (N=68)	48.8±17.8	51.5 (18, 80)

**Table 3.** LMA size and expected outcome by LMADiff status.

Variables	LMADiff		p-value
	False (LMADiff=0) N=52	True (LMADiff=1) N=17	
**LMA Size**, n (%) 3,4 5	n=27 18 (66.7) 9 (33.3)	n=7 3 (42.9) 4 (57.1)	0.387
**No of Attempts** 1 >1	n=29 28 (96.6) 1 (3.5)	n=4 1 (25.0) 3 (75.0)	0.003
**Ideal size by weight** 3,4 5	n=50 15 (30.0) 35 (70.0)	n=17 6 (35.3) 11 (64.7)	0.684
**Ideal size by height** 3,4 5	n=48 28 (58.3) 20 (41.7)	n=17 14 (82.4) 3 (17.7)	0.087
**ExpecDMV**, n (%)	(11.5)	3 (17.7)	0.679
**ExpecDLMA**, n (%)	4 (7.7)	2 (11.8)	0.631
**ExpecDL**, n (%)	11 (21.2)	10 (58.8)	0.003
**ExpecDI**, n (%)	7 (13.5)	3 (17.7)	0.699
**ExpecDSA**, n (%)	1 (1.9)	4 (23.5)	0.012

Expec: predicted, expected, at airway assessment; DMV: difficult mask ventilation; DLMA: difficult Laryngeal Mask Airway; DL: Difficult Laryngoscopy; DI: Difficult Intubation; DSA: Difficult Surgical Airway

**Table 4.** Summary statistics for LMADiff and LMASuccess.

Outcome	Frequency (percentage) N=69
LMADiff 0 1	52 (75.4) 17 (24.6)
LMASuccess 0 1	2 (2.9) 67 (97.1)

**Table 5.** Two independent predictors of LMA difficulty.

Predictor	β Coefficient	Standard Error	*P* value	Adjusted odds ratio (95% Confidence Interval)
Female	1.466	0.723	0.043	4.33 (1.05, 17.85)
Neck>=44	1.810	0.787	0.021	6.11 (1.31, 28.56)

**Table 6.** Diagnostic value of the cut-off for the number of risk factors in predicting a difficult mask ventilation.

Cut-off for number of risk factors	Sensitivity	Specificity	Likelihood ratio positive	Likelihood ratio negative	Positive predictive value	Negative predictive value
1	0.875	0.500	1.75	0.25	0.359	0.926
2	0.063	0.980	3.50	0.956	0.500	0.766

Likelihood ratio positive=Sensitivity/(1-Specificity) Likelihood ratio negative=(1-Sensitivity)/Specificity The table displays the sensitivity and specificity if we use the given value of the number of risk factors possessed by patients as a cut-off to classify LMA difficult. For example, when we use number of risk factors at 1 as a cut-off, i.e., any patients with >=1 risk factors will be classified as LMA Diff=1 and any patients with <1 risk factors will be classified as LMA Diff=0, the sensitivity will be 0.875 and specificity will be 0.500.

## Discussion

In the present investigation, risk factors in 69 LMA primary airway management placements were assessed. The incidence of difficult LMA placement in our study was 24.6% and the LMA failure rate was 2.9%. Moreover, the incidence of failed LMA placement in our study is consistent with previous studies ^9, 13, 18, 21, 22^, ranging from 0.19 to 4.7%.

Although from a large database, the study resulted only in a few placements, which is consistent with the practice of our teaching academic center and that could give a possible explanation to the increased incidence of difficult LMA placement in our study. Beside the limited number of uLMAs utilized electively, the study provides an interesting perspective on predictive factors pertaining laryngeal mask placement: indeed, two independent risk factors were found, neck circumference ≥44 cm and female gender. A predictive score that would assist the clinician in identifying difficult LMA placement was also developed, resulting in a model with low sensitivity but specificity of 98% and a negative likelihood ratio of 95.6% (for instance, excluding difficult LMA placement in male patients with neck circumference <44 cm).

The current study supports previous findings regarding the correlation of obesity and difficult airway ^23– 26^, since increased neck circumference is also an independent risk factor for difficult mask ventilation (DMV) and difficult intubation. The most interesting finding of this study is that female gender, rather than male gender is associated with difficult LMA placement in this study population. In contrast, Ramachandran *et al.* found that male gender was a predictive factor for failed LMA placement ^13, 18^.

Age distribution of our population was considered as a cause for this difference. Indeed, age distribution of our female population could be associated with an increased proportion of postmenopausal women. Previous studies have demonstrated that the prevalence and severity of Obstructive Sleep Apnea (OSA) is increased in postmenopausal women, as compared to pre-menauposal women, which may be related to functional changes ^27^. However, history of OSA was not an independent predictive factor in our population. This can be attributed to the retrospective nature of our study, where OSA assessment was assessed only by patient history. Of interest, a recent but unpublished study has highlighted that the female gender was a predictor for difficult LMA placement in a study population of more than 400 patients, where LMA placement was performed by a single skilled clinician ^28^.

Of the other airway variables that were evaluated in our study, none was identified as an independent predictor of LMA failure: this finding differs from that of Ramachandran *et al.*, who recognized the absence of teeth as an independent predictor of LMA failure, and the differences could be attributed to population included in the two studies, particularly the limited number of outcomes of our study, possible underutilization of the LMA as a primary airway device, as compared to other airway devices, the increased incidence of difficult LMA placements in our population, and the placements by trainees. Discussing the limitations of the present investigation, it is necessary to mention the retrospective nature as well the stepwise selection that may contribute to bias the study, and the subjective nature of the definition of difficult LMA placement. Additionally, we assumed that all anesthesiology residents had similar educational skills based on a previous study ^19^, which also could have affected our findings.

In conclusion, two risk factors for LMA placement difficulty were identified: female gender and large neck circumference. Considering the airway as an entity, neck circumference is being increasingly recognized as a significant predictive factor for difficulty with airway management, especially when it is considered across the spectrum of difficulties.

## Data availability

The data referenced by this article are under copyright with the following copyright statement: Copyright: © 2015 Katsiampoura AD et al.

Data have been obtained from databases at the Memorial Hermann Hospital, Texas Medical Center, Houston, IRB approval HSC-MS-07-0144. The author can support applications to the Institutional Board to make the data accessible upon individual request. Please forward your requests to Davide *Cattano*.
