# Supplementary material for: Laryngeal mask placement in a teaching institution: analysis of difficult placements
Source: F1000Res. 2015 Apr 29;4:102. [Version 1] doi: 10.12688/f1000research.6415.1 (PMC4566281; doi:10.12688/f1000research.6415.1)
Supplement: Peer review history

# Peer review: Referee response for version 1

**DOI:** 10.5256/f1000research.6882.r9937

**Published:** 2015-09-08

**Authors:** Massimo Micaglio

**Affiliations:** 1 Department of Anesthesia and Intensive Care, Careggi University Hospital, Florence, Italy

I read with interest this investigation and I wish to thank the Authors for the request of my review.

With this retrospective study they aimed to identify a simple, objective list of predictive factors for difficult laryngeal mask airway placement. As they underlined, at present such a list is not available.

The topic is up-to-date, remarkable and well represents a growing body of research on EGA.

Because LMA placement was performed by residents, some well known factors contributing to difficult placement of a LMA (type of anesthesia induction, dose of hypnotic agents, proper “waiting time” before LMA insertion, NMBA usage or not, etc.), could have been considered. But some limitations of the study are correctly stated by authors.

The conclusions that large neck circumference and female gender were independently associated with difficult LMA placement definitely provide data potentially useful to clinicians.

I have read this submission. I believe that I have an appropriate level of expertise to confirm that it is of an acceptable scientific standard.

# Peer review: Referee response for version 1

**DOI:** 10.5256/f1000research.6882.r8512

**Published:** 2015-06-23

**Authors:** Massimiliano Carassiti

**Affiliations:** 1 Department of Anaesthesia and Intensive Care, School of Medicine, Campus Bio-Medico University of Rome, Rome, Italy

The major limitation of the study is the small sample size and that the degree of experience of the operators is not classified.

The study identifies the circumference of the neck and the female gender as independent risk factors for the positioning of the LMA. The "female risk" is in contrast to the study of Ramachandran *et al.*, which had a larger sample size and included the experience of operators, and this must be stressed more effectively in discussion

I have read this submission. I believe that I have an appropriate level of expertise to confirm that it is of an acceptable scientific standard.

# Response

**Published:** 2015-06-24

**Authors:** Davide Cattano

**Affiliations:** Department of Anesthesiology, The University of Texas Medical School at Houston, USA

We want to thank Dr Carassiti comments. The experience of the operators is definitely important and a major factor determining the success of a device or technique. In the specific case of the current investigation, which was derived retrospectively from a larger database, all airway manipulation/management were carried out by trainees (CA-1-CA3) with a minimum experience of six month (meaning the CA1 class was enrolled only after their first 6 months of training were elapsed), and one to one supervised by an attending anesthesiologist, which ultimately was responsible. We believe the point raised are extremely important, yet relying on the fact that tailoring for every single case to the experience based, would require a significant larger sample. While we evaluated the cases by experience of the operators, in general, we did not find any skewed distribution, yet a case by case was not deemed important, because of the number of cases. As we mentioned in our discussion the results have to be carefully considered observationally, with the possibility of selection bias due to the nature of the "mother" study to start with, to the limited number of cases, that would include a selected group of individuals (half of the residents, because they were assigned to a full airway assessment on multiple testing), yet not for any particular reason, different. Ramachandran *et al.*, accessed a single institution, residency program database, were, otherwise, it is reasonable to think the majority of EGA placements occurred by experienced staff. Yet the sample is much larger to question our findings. An interesting hypothesis, based on both studies, is that a differential complexity is determined by oro-pharyngeal anatomy vs laryngeal structures and neck morphology. That may explain that in males, a larger LMA would have chosen based on weight, while oropharyngeal or laryngeal structures would not accommodate for instance a size 5. In our overweight/obese population that may also explain why females patients (maybe largely selected for LMA because not as "morbidly obese", or accidentally clustered in our sample) resulted in more failures in our cohort.

It is a great opportunity, as suggested by Dr Vannucci, to identify new frontiers of EAD research.

# Peer review: Referee response for version 1

**DOI:** 10.5256/f1000research.6882.r8514

**Published:** 2015-05-12

**Authors:** Andrea Vannucci

**Affiliations:** 1 Department of Anesthesiology, Washington University in St. Louis, St. Louis, MO, USA

I wish to thank the authors very much for the opportunity to review this interesting investigation of theirs.

In this retrospective study, the authors correlated a set of prospectively collected data including a comprehensive assessment of upper airway and neck anatomy and function to the event " *difficult laryngeal mask (LMA) placement*" in 2,348 adult patients. Those patients, who underwent elective, non-obstetric procedures, received an LMA Unique™ as the primary device to control the airway during their surgery.

LMA placement was generally performed by anesthesiology residents.

Difficult LMA placement was defined as “ *… either inability to physically place a LMA device or inadequacy of ventilation, oxygenation, or airway protection after placement that required conversion to an alternative technique.*”

The authors identified 69 patients that met their criteria for “difficult LMA placement”.

By logistic regression analysis, they came to the conclusion that neck circumference larger than 44 inches and female sex were independently associated with difficult LMA placement.

The hypothesis of the study that a comprehensive preoperative assessment of the airway may help predicting difficulties at the placement of an LMA is an interesting one, certainly worth of an exploration.

The main issue I see in the abstract, title, methods and conclusion of the study is an ambiguous definition of “difficult LMA placement” that likely includes two separate entities: a) failure to position an LMA (2 cases in the study, as per results presented in Table 4); and b) failure of the LMA during the use (15 cases, again as per Table 4 after removing the two above mentioned failures). 

If my above interpretation of the study premises is correct, it is unlikely we can understand the causes of intraoperative failure of the LMA if intraoperative factors are not explored.

In particular, I believe that it would be important to consider what procedures the patients were undergoing when the LMA failure was detected, how much after the induction of the anesthesia and the start of the surgery the failure occurred, and other intraoperative and anesthetic factors like position of the operating table (flat, Trendelemburg, reverse Trendelemburg, etc.), the type of anesthesia (inhalational, intravenous, balanced technique, etc.), and so on.

In fact, intra-operative factors may have had a more relevant role in determining the intra-operative failure of the LMA than the baseline patient anatomical characteristics.

This additional information could provide more insight on the mechanisms of LMA failure and could also facilitate a comparison of the results of this study with the ones obtained by Dr. Ramachandran and colleagues at the University of Michigan.

Therefore, my advice to the authors is to add as much information as they can retrieve in their database on intraoperative factors that may have had a role in determining LMA failure.

Besides this significant limitation, the study approaches the problem of "LMA failure" (either initial failure to position or later failure during surgery) from an interesting perspective and provides some new and interesting data that can be of interest to many clinicians.

A few additional questions:

- Table 1: last row title “Thyroid”. What do the authors mean here?
- Table 3: Can the authors provide the specific references for each of the categories: “ExpecDMV”, “ExpecDLMA”, “ExpecDL”, “ExpecDI”, “ExpecDSA”?
- Methods: did the authors retrieve (part of) their data from an electronic or a paper anesthetic records? This information could be reported in the methods section.
- Discussion, page 6. Can the authors further clarify the following point (in particular what they intend by "stepwise selection" and how this relates to the bias of the study) “Discussing the limitations of the present investigation, it is necessary to mention the retrospective nature as well the stepwise selection that may contribute to bias the study, and the subjective nature of the definition of difficult LMA placement.”?

I have read this submission. I believe that I have an appropriate level of expertise to confirm that it is of an acceptable scientific standard, however I have significant reservations, as outlined above.

# Response

**Published:** 2015-05-12

**Authors:** Davide Cattano

**Affiliations:** Department of Anesthesiology, The University of Texas Medical School at Houston, USA

We are very grateful to Dr Vannucci for his timely and well thought review. We appreciate the opportunity to respond to some of his comments and clarify few of the work's points.

The study we extracted the LMA information was designed to evaluate the prediction of the difficult airway (ref 19). It was based on a paper-based evaluation form that contained all 11 of the predictive factors the ASA and other professional societies recommended (based on the practice management guidelines from 2003) as well as others. As airway management progressed during the study, different airway options populated our database (paper form collected), including LMA placements. We agree that anatomy is not the only factor contributing to difficult or failed placement of an EAD (proper relaxation, proper technique) and others may related to anatomy though, like sizing (see in the tables the estimation of used size versus optimal size). Other than placement, other factors contributes to overall failure of an EAD, which aim to be a ventilation conduit and patent airway assistance. So ventilatory mechanical and non mechanical issues can arise. Dr Vannucci correctly suggest that such factors (intraoperative) would be of great interest. Because of the scope and information available we limited to the factors contributing to the difficulty or failure of initial positioning and ventilation. The anesthesiologist attending decided and reported whether or not the LMA was satisfactory and the assessment of success (yes, no, i.e. failed) and easiness (yes, no, i.e. difficult and easy). So of the 69 placements, 17 were considered difficult, but not failed, and of the 17 also 2 were considered ultimately a failure (that is the meaning of the 2 separate tables). Expected DMV, DI etc, are based on the preoperative definition of the airway assessment, meaning the airway evaluation resulted in a predicted difficult bag mask ventilation, difficult laryngoscopy, difficult intubation etc. They not necessarily meant a difficult airway, but the predictive portion. The Thyroid in the first table was the comment of resident evaluation or acknowledging a patient condition significant for "thyroid pathology" (could have been goiter as well as clinical diagnosis of hyper or hypothyroidism, aspecifically).

Last, the stepwise regression was performed by our statistician taking into account the primary univariate and multivariate analysis. Considering the small sample we acknowledged that study limitations need to be taken into account when interpreting the results.

In conclusion the current results propose that there may be unrecognized anatomical factors, maybe related to sizing methods, which the authors are also evaluating in other investigations (13, 17). However Dr Vannucci's points pertaining the value of post placement onset ventilation and seal failures are in need of further exploration.

# Response

**Published:** 2015-05-12

**Authors:** Andrea Vannucci

**Affiliations:** Washington University in St. Louis, USA

Thank you to the authors for the clarifications and additional information they provided. I am fine with their response.
